# Multicentric Case Series and Literature Review of Coccidioidal Otomastoiditis

**DOI:** 10.3201/eid2907.230129

**Published:** 2023-07

**Authors:** Ilan S. Schwartz, Caitlyn Marek, Harleen Sandhu, Ahmed Abdelmonem, Greti Petersen, Emma Dishner, Arash Heidari, George R. Thompson

**Affiliations:** Duke University School of Medicine, Durham, North Carolina, USA (I.S. Schwartz);; Royal Inland Hospital, Kamloops, British Columbia, Canada (C. Marek);; Kern Medical Center, Bakersfield, California, USA (H. Sandhu, G. Petersen, A. Heidari);; Valley Fever Institute, Bakersfield (H. Sandhu, A. Heidari);; Baylor University Medical Center, Dallas, Texas, USA (A. Abdelmonem, E. Dishner);; UC-Davis Medical Center, Davis, California, USA (G.R. Thompson III)

**Keywords:** coccidioidomycosis, fungi, lung diseases, respiratory infections, fungal, coccidioidomycosis, otomycosis, coccidioidal otomastoiditis, United States, Canada

## Abstract

Coccidioidomycosis involving the ear, mastoid bone, or both is uncommon. We describe 5 new cases from the United States and review 4 cases reported in the literature of otomycosis and mastoiditis caused by *Coccidioides*. Of the 9 cases, 8 were linked to residence in or travel to California. Two patients had poorly controlled diabetes mellitus, 7 had otomastoiditis, 1 had otitis externa without mastoid involvement, and 1 had mastoiditis without otic involvement. Four patients had concurrent or prior pulmonary coccidioidomycosis. Ipsilateral facial nerve palsies developed in 2 patients. All patients received antifungal treatment for varying durations, and 8 of the 9 patients underwent surgical debridement. Clinicians should consider coccidioidomycosis as a differential diagnosis for otomastoiditis in patients with geographic risks.

The clinical manifestations of coccidioidomycosis, an expanding endemic mycosis caused by *Coccidiodes immitis* and *C. posadasii* (*1*), are notoriously protean (*2*,*3*). Infection occurs primarily by inhalation of aerosolized arthroconidia, which undergo morphologic change within the lungs and turn into spherules (large structures containing endospores) (*4*). Coccidioidomycosis is a highly variable illness and may be asymptomatic or cause a mild respiratory illness in up to 60% of infected persons and an uncomplicated pulmonary infection in most others (*4*). Dissemination or progressive pulmonary disease affects 1%–2% of persons infected with *Coccidioides* spp. (*2*). In those persons, after spherule rupture, endospores may spread hematogenously or through the lymphatic system to virtually all organs, although extrapulmonary clinical disease at sites other than the brain, skin, bone, or psoas muscle is uncommon (*5*). Those infrequently encountered sites of disease may present diagnostic and therapeutic challenges (*2*).

Coccidioidomycosis involving the middle or outer ear, mastoid bone, or both is uncommon. We describe 5 cases of otomycosis and mastoiditis caused by *Coccidioides* spp. in the United States and review 4 cases reported in the literature. Institutional Review Board ethics approval was obtained at Kern Medical Center (Bakersfield, California, USA) and was not required for unidentified case reports at the other medical centers involved.

## Cases

### Case 1

In 2019, a 76-year-old White man from California sought care for left-sided hearing loss that started after an eschar developed on the left tragus and cheek. He had no history of trauma. An otolaryngologist noted a middle ear effusion, which was managed conservatively with topical therapies and tympanostomy tube insertion. Because the effusion persisted despite those interventions, the otolaryngologist sent samples for microbiological analysis, including bacterial and fungal cultures. Bacterial cultures were negative; however, after 3 days of incubation, a mold was isolated, identified by DNA Probe (Hologic, Inc., https://www.hologic.com) as *Coccidioides* spp. A chest radiograph was unremarkable. Oral fluconazole (400 mg/d) was started. Although the effusion decreased, hearing did not improve. After 3 months of therapy, fluconazole was discontinued because of a widespread maculopapular exanthem. Two months thereafter, the unilateral hearing loss persisted. At that time, computed tomography (CT) of the head showed complete opacification of the left mastoid air cells, a soft tissue infiltrate within the middle ear chambers involving the epitympanic recess, and thinning of the left mastoid bone without obvious bony destruction or intracranial extension. Otoscopic examination revealed extensive debris and discharge throughout the ear canal and tympanic membrane perforation. The ear canal was debrided biweekly for 3 months, which led to healing of the membrane and decreased erythema and discharge from the ear canal. Subsequent treatment was itraconazole (200 mg by mouth 2×/d for 6 months). The patient’s hearing partially improved after several weeks of antifungal treatment; improvement reached a plateau at 5 months but did not return to baseline. At the end of therapy, the patient was no longer available for follow-up.

### Case 2

In 2015, a 52-year-old man from California sought care for headache and jaw pain. Findings from a physical examination, including the oropharynx and tympanic membranes, were unremarkable. Initially, nonsteroidal antiinflammatories were prescribed, and the condition was managed expectantly. Over the next 4 months, the intensity of the patient’s symptoms increased and the pain localized to the left mastoid. Magnetic resonance imaging (MRI) demonstrated left mastoiditis. There was no clinical or radiographic evidence of pulmonary involvement. A biopsy sample was collected from the mastoid; bacterial and fungal cultures were negative, but *Coccidioides* spherules were identified by histopathologic examination. Subsequently, serologic testing demonstrated a *Coccidioides* complement fixation (CF) titer of 1:8, and *Coccidioides* immunodiffusion was positive for IgG. Treatment with oral fluconazole (400 mg/d) was initiated. At a follow-up visit 2 years later, the patient had residual mastoid pain but no other signs or symptoms of disease, and his CF titer had decreased to 1:2. Repeated imaging after 26 months revealed substantial improvement, but radiographic evidence of mastoiditis persisted. Fluconazole was continued indefinitely, although the patient has since been unavailable for follow-up.

### Case 3

In 1999, a 42-year-old White man from California sought care for right ear fullness and tinnitus. Examination indicated a right middle ear effusion without erythema. Systemic antihistamines were prescribed; however, the patient returned 1 month later without clinical improvement. Sequential empiric courses of oral prednisone (20 mg/d for 14 days) and amoxicillin/clavulanate (500/125 mg 3×/d for 10 days) also did not lead to improvement. Soon thereafter, ipsilateral facial nerve palsy developed. MRI revealed evidence of an ongoing middle ear effusion and mastoiditis. Tympanocentesis was performed, and *C. immitis* was identified on cultures of the aspirated middle-ear fluid. Serum *Coccidioides* CF titer was 1:4, and *Coccidioides* immunodiffusion was positive for IgG. After further questioning, the patient recalled having had an episode of subacute pneumonia ≈18 months earlier that did not respond to levofloxacin. After *Coccidioides* otomastoiditis was diagnosed, oral fluconazole (800 mg/d) was prescribed. Treatment was continued for 3 years, the ear effusion and tinnitus resolved, the 7th nerve palsy partially improved, the CF titer declined to undetectable, and the radiographic appearance of the mastoiditis improved. After 3 years, the patient self-discontinued fluconazole. He has remained asymptomatic with undetectable CF titers through 21 years of follow-up.

### Case 4

**Figure 1:**
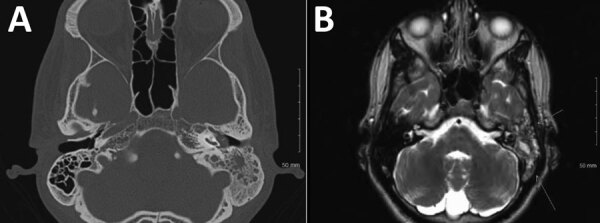
Radiographic findings from 22-year-old Hispanic man from California, USA (case 4), in multicentric case series of coccidioidal otomastoiditis. A) Computed tomography scan of the head, showing opacification of the mastoid. B) Magnetic resonance image of brain, showing mastoiditis.

In 2014, a 22-year-old Hispanic man from Bakersfield, California, in the *Cocciodioides* epicenter of San Joaquin Valley, who had a medical history of uncontrolled diabetes mellitus type 1, received a diagnosis of pulmonary and osteoarticular coccidioidomycosis but was nonadherent to therapy with fluconazole and then posaconazole (substituted because of liver injury). Peak CF titer was 1:256. After 1 year of nonadherence, he sought care for left ear pain and purulent drainage for 5 days. Symptom onset was gradual, constant, aching, and sharp, with radiation to left jaw and left eye. Left otitis externa was diagnosed and treated with oral amoxicillin and topical neomycin, polymyxin B sulfates, and hydrocortisone otic solutions. He was unavailable for follow-up for another 6 months, at which point he sought care for progressive left-sided hearing loss of 1 week’s duration and diffuse pounding headache, nausea, and vomiting for 1 month. Neurologic examination suggested left conductive hearing loss. Results for other cranial nerves were unremarkable. Otoscopic examination revealed that the left tympanic membrane was erythematous and bulging with a middle ear effusion. Results of lumbar puncture were unremarkable. *Coccidioides* CF at this time was 1:64. CT showed complete opacification of the mastoid air cell system on the left side with fluid in the middle ear (Figure 1, panel A). MRI showed left mastoiditis with an extradural collection of fluid. The patient underwent a left myringotomy with insertion of a tympanostomy tube. Ten days postoperatively, he remained symptomatic, reporting a constant headache radiating from his left eye to the occipital area, along with light-headedness and left ear hearing loss. Repeat CT imaging 2 weeks after the myringotomy suggested persistent left otomastoiditis. MRI showed heterogenous signal in the left mastoid and middle ear and discontinuity of the roof of the left mastoid but no temporal lobe or middle cranial fossa involvement (Figure 1, panel B). Because of the lack of response to tympanostomy tube placement, a left mastoidectomy and tympanoplasty were performed. Mastoid tissue was cultured, and *C. immitis* grew after 28 days. The patient was hospitalized, and a 6-week course of intravenous liposomal amphotericin B monotherapy was initiated, which he continued as an outpatient. At 6-week follow-up, his hearing had gradually increased. Antifungals were changed to posaconazole (400 mg/d orally). One year later, *Coccidioides* CF had improved to 1:8, and the patient remained well.

### Case 5

**Figure 2:**
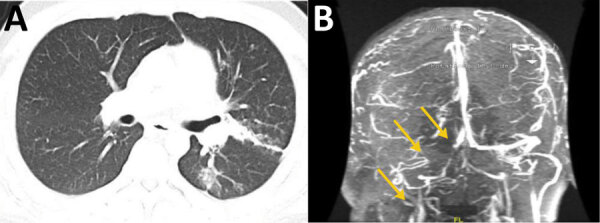
Radiographic findings from 25-year-old Hispanic man from New Mexico, USA (case 5), in multicentric case series of coccidioidal otomastoiditis**.** A) Computed tomography scan of the chest with contrast, showing septic emboli of the left lower lung lobe and lingula with developing pneumonia. B) Magnetic resonance venogram, showing stable thrombus of the right jugular bulb, right sigmoid and transverse sinuses, and partially occlusive thrombus in the anterior superior sagittal sinus, seen by lack of enhancement (arrows) compared with the left side.

In 2021, a 25-year-old Hispanic man from New Mexico with a past medical history of poorly controlled diabetes mellitus type 2 sought care in Texas for right ear pain. Physical examination indicated swelling and tenderness of the right mastoid, along with purulent drainage from the right ear. CT of the temporal bone showed acute right otomastoiditis with thrombophlebitis of the right transverse and sigmoid dural sinuses and right internal jugular vein. The patient also had an associated venous thrombus and abscess under the right mastoid process. He underwent a right mastoidectomy and myringotomy tube placement for his abscess. The abscess cultures grew methicillin-susceptible *Staphylococcus aureus* and later, after the patient was discharged, *Coccidioides*. The patient was discharged with a prescription for intravenous nafcillin for 6 weeks and oral apixaban. However, the abscess and thrombus persisted, and he began to experience shortness of breath 1 month after discharge. He was hospitalized because of disease progression, and chest CT showed septic emboli of the left lower lung lobe and lingula (Figure 2, panel A). Given the lack of improvement, it was thought that the *Coccidioides* infection was playing a larger role in the disease, and intravenous liposomal amphotericin B (5 mg/kg) and oral fluconazole (800 mg/d) were initiated. The right neck abscess was incised and drained, and cultures grew *Coccidioides*. CF titers were 1:32. Magnetic resonance venography showed progression of the thrombus to the sagittal sinus despite anticoagulation therapy. The interventional radiologist attempted a thrombectomy, which was unsuccessful. It was determined that the thrombus progression was most likely caused by *Coccidioide*s thrombophlebitis, and a heparin infusion was started. At 6-week follow-up, the patient was improving with no neurologic dysfunction; repeat magnetic resonance venography showed stability of the thrombus (Figure 2, panel B), and the patient was discharged with prescriptions for voriconazole and apixaban. Repeat CF titers were 1:64 and remained stable for 10 months.

## Literature Review and Synthesis

With a literature search, we identified 4 additional cases of coccidioidomycosis involving the ear, mastoid bone, or both (*6*–*8*) and summarized them along with the 5 cases in our series (Table). Eight patients for whom details were available had lived in or traveled to California. Ages ranged from 4 to 76 years, and 6 of 9 were male. Ethnicity was known for 7 patients: 4 were Hispanic and 3 were White. Two patients had poorly controlled diabetes mellitus (1 each with types 1 and 2). Another patient had systemic lupus erythematosus, which had been treated with corticosteroids 1 month before the coccidioidal otomastoiditis developed. In retrospect, however, misdiagnosed coccidioidomycosis was probably the cause of the fatigue, fever, and arthralgias (with positive antinuclear and anti-DNA antibodies) attributed to lupus. Seven patients had otomastoiditis, 1 had mastoiditis without otitis, and 1 had otitis without mastoiditis. Ear pain was noted for 5 patients, mastoid swelling or tenderness for 3, and hearing loss for 2. Two patients had cutaneous lesions at the ear.

**Table:** Summary of cases of coccidioidomycosis involving the middle or outer ear or mastoid detected in multicentric case series of coccidioidal otomastoiditis, California, USA, and literature review of other cases*

Case	Ref	Age, y/sex; race/ethnicity	Comorbidity	Syndrome	Symptoms	Diagnosis	Management	Outcome
1	(*6*)	23/F; Hispanic	Had been diagnosed with systemic lupus erythematosus 1 mo earlier on basis of fatigue, fever, arthralgias, proteinuria, and positive antinuclear antibodies, and treated with corticosteroids; in retrospect, sign/symptoms were probably caused by coccidioidomycosis.	Otitis externa → otitis media, mastoiditis	L ear pain, fever. After several months of antifungal therapy, partial L facial nerve paralysis developed.	Middle ear fluid culture grew *Coccidiodes. immitis*; CF 1:8.	Mastoid atticotomy, irrigation with amphotericin B (continued 3 weeks after final surgery); facial nerve decompression and temporal bone debridement, followed by IV amphotericin B for 5 d, followed by miconazole for at least 3 mo; irrigation of ear canal with amphotericin B (2 g) over 3 mo, including 3 weeks after final curettage	Good clinical response, with return of function to facial nerve almost entirely in all branches. No relapse through 1 y of follow-up.
2	(*6*)	43/M; White	None	Pulmonary and lymph node disease initially; otomastoiditis 1.5 y later	R ear pain and a “squishy” sensation	Histologic diagnosis of coccidioidomycosis from lymph node; *C. immitis* cultured from middle ear fluid. *Coccidioides* CF 1:4.	Tympanoplasty and mastoidectomy, myringotomy and revision tympanoplasty, grommet placement; local irrigation of mastoid with amphotericin B, systemic amphotericin B (267.5 mg IV for 7 d)	Drainage subsided by 5 mo. No evidence of disease recurrence at 1 y.
3	(*7*)	20; Hispanic	None	Otitis externa	Cutaneous lesion on external ear and periauricular skin	Histopathologic examination of skin biopsy specimen demonstrated spherules of *Coccidioides*.	Fluconazole (400 mg/d orally for unknown duration); frequent debridement of ear canal	Unknown
4	(*8*)	4/F; unknown	None	Otomastoiditis; incidental left lower lobe lung cavity.	6-mo history of R ear pain, mild hearing loss, intermittent fever; swelling behind R ear	Histopathologic examination of mastoid biopsy demonstrated spherules of *Coccidioides*; biopsy of same grew *C. immitis*.	Mastoidectomy; amphotericin B (IV) for 6 wk	No recurrence (timeline not stated). Serial decrease in *C. immitis* antigens.
5	This study (case 1)	76/M; White	None	Otomastoiditis	Cutaneous lesion over L tragus and cheek, L hearing loss	*C. immitis* cultured from middle ear fluid.	Fluconazole (400 mg/d orally for 3 mo); debridement; itraconazole (200 mg 2×/d orally for 6 mo)	Persistent hearing loss after 6 mo of follow-up.
6	This study (case 2)	52/M**; **unknown	None	Mastoiditis	Headache and jaw pain	Histopathologic examination of mastoid biopsy demonstrated spherules of *Coccidioides.* CF titer 1:8; ID positive for IgG.	Fluconazole (400 mg PO daily for 26 mo), then no longer available for follow-up	Residual pain and ongoing radiographic evidence of mastoiditis after 26 mo of therapy.
7	This study (case 3)	42/M; White	None	Pneumonia, followed 18 mo later by otomastoiditis	R ear fullness and tinnitus; later ipsilateral facial nerve palsy developed	*Coccidioides* cultured from middle ear fluid. CF titer 1:4; ID positive for IgG	Fluconazole (800 mg/d orally for 3 y)	Resolution of ear effusion and tinnitus, partial resolution of facial palsy, radiographic improvement, CF titer decreased to undetectable. Well in follow-up with negative CF titers for 21 y.
8	This study (case 4)	22/M; Hispanic	Diabetes mellitus type 1	Pulmonary coccidioidomycosis →osteoarticular coccidioidomycosis→ otomastoiditis	Left ear pain, purulent drainage, hearing loss, headache, nausea, and vomiting	*C. immitis* cultured from mastoid biopsy. CF titer 1:256	Otomastoiditis developed after poor adherence to fluconazole (800 mg); mastoidectomy and tympanoplasty, followed by liposomal amphotericin B (IV) for 6 wk, followed by posaconazole (400 mg/d orally) for several months before patient was no longer available for follow-up	Clinical improvement. Gradual return of hearing. CF titer decreased to 1:8. Long-term follow-up data unavailable.
9	This study (case 5)	25/M; Hispanic	Diabetes mellitus type II	R otomastoiditis→R internal jugular vein thrombus and dural venous thrombus→ septic emboli	R ear pain, purulent drainage, R mastoid tenderness and shortness of breath	*Coccidioides* cultured from mastoid tissue (along with *Staphylococcus aureus)* and later a neck abscess. CF titers 1:32	Mastoidectomy and myringotomy tube placement, followed by liposomal amphotericin B (IV) and fluconazole (800 mg/d orally); heparin infusion for thrombosis	Clinical improvement, pending follow-up imaging to determine regression of dural venous thrombus

*CF., complement fixation; ID, immunodiffusion; L, left; R, right; ref, reference.

In addition to the ears or mastoids, 4 patients had previous or concomitant pulmonary disease consistent with coccidioidomycosis, including 1 patient who additionally had osteoarticular coccidioidomycosis. Ipsilateral facial nerve palsies developed in 2 patients, early for one and several months into treatment for the other. In 1 patient, coccidioidal otomastoiditis was complicated by ipsilateral internal jugular thrombosis and septic emboli.

*Coccidioides* species was cultured from middle ear fluid for 4 patients, mastoid tissue for 2, and both middle ear fluid and mastoid for 1. For 3 patients, diagnosis was based on histopathologic findings of spherules in mastoid tissue and for another, by histopathologic examination of skin. All 9 patients received antifungal drugs: amphotericin B for 2, amphotericin B followed by an azole for 3, and azoles alone for 4. Eight patients underwent surgical irrigation and debridement.

## Discussion

Coccidioidomycosis is one of the most common dimorphic fungal diseases encountered in North America; the geographic range on the continent is primarily the southwestern United States and parts of Mexico (*9*). Myriad manifestations have been described (*4*). Ear or mastoid bone involvement, however, is rare with coccidioidomycosis. In addition to the 5 cases in our series, we identified only 4 cases of ear or mastoid coccidioidomycosis in the literature. In a database of 3,000 patients with coccidioidomycosis at Kern Medical Center, only 1 case of otomycosis or otomastoiditis (case 4) was identified, highlighting the uncommon nature of this manifestation.

The mechanism by which coccidioidomycosis involves the ear or mastoid is unclear. Because those structures are contiguous with the upper respiratory tract, primary infection of the ears and mastoids after inhalation of *Coccidioides* arthroconidia is plausible. However, there was evidence of pulmonary disease for only 4 of 9 patients, consistent with the lack of pulmonary signs or symptoms in other patients with disseminated coccidioidomycosis involving sites not contiguous with the airways (*10*). Hematogenous dissemination is also a potential mechanism; Scalarone and Huntington showed in a murine model of coccidioidomycosis that intraperitoneal inoculation (which mimics hematogenous infection) with a low-virulence strain of *C. immitis* can lead to infection that localizes to inner ear structures and mastoids (*11*). It is unclear whether strain virulence or inoculation route differs among patients with this uncommon manifestation.

Although most cases of otitis or mastoiditis are bacterial, the diagnosis of coccidioidomycosis should be considered for patients with otitis or mastoiditis who have resided in or traveled to areas of risk for coccidioidomycosis, especially if the case does not respond to empiric antibacterial therapy. The diagnosis can be suggested by serologic testing, but confirmation requires culture of discharge from the ear or by biopsy of the mastoid for histopathology and fungal culture.

The optimal treatment for coccidioidomycosis involving the ear or mastoids is unknown. Most of the patients we reviewed underwent debridement, as is typically performed for fungal ear or mastoid disease. Antifungal drugs are a major component of management, although the optimal agent and duration remain unresolved. In this series, the duration of therapy ranged from 1 week (of amphotericin B) to indefinitely (suppressive azole therapy), and the optimal duration is unresolved. Repeated clinical, radiographic, and serologic evaluation with CF should be obtained before therapy is discontinued. Given the proximity and potential for invasion of the central nervous system, lifelong azole therapy should be weighed against toxicities associated with long-term use (*12*). Our patient series demonstrates the protean nature of coccidioidomycosis. Clinicians should consider a diagnosis of coccidioidomycosis for patients with otomastoiditis refractory to standard therapy and a history of exposure to areas of geographic risk.
